# Determination of porphyrins in oral bacteria by liquid chromatography electrospray ionization tandem mass spectrometry

**DOI:** 10.1007/s00216-015-8864-2

**Published:** 2015-07-14

**Authors:** Jonas Fyrestam, Nadja Bjurshammar, Elin Paulsson, Annsofi Johannsen, Conny Östman

**Affiliations:** Department of Environmental Science and Analytical Chemistry, Analytical and Toxicological Chemistry, 106 91 Stockholm, Sweden; Department of Dental Medicine, Karolinska Institutet, 141 04 Huddinge, Sweden; Department of Aquatic Sciences and Assessment, Swedish University of Agricultural Sciences, 750 07 Uppsala, Sweden

**Keywords:** HPLC/MS/MS, *Aggregatibacter actinomycetemcomitans*, *Porphyromonas gingivalis*, *Saccharomyces cerevisiae*, Porphyrins, Oral bacteria

## Abstract

Biofilms in the oral cavity can be visualized by fluorescence and a common assumption is that the endogenously produced porphyrins in certain bacteria give rise to this fluorescence. Porphyrin content in oral bacteria has been sparingly investigated, and non-selective detection techniques such as utilizing the Soret fluorescence band of porphyrins are often used. In the present study, a quantitative and selective method for the determination of porphyrins in oral bacteria has been developed and validated using high performance liquid chromatography-tandem mass spectrometry. Lysis of bacteria using Tris-EDTA buffer together with ultrasonication showed high microbial killing efficiency ≥99.98 %, and sample clean-up using C_18_-solid phase extraction resulted in low matrix effects ≤14 % for all analytes. Using this method, the porphyrin content was determined in the two oral pathogens *Aggregatibacter actinomycetemcomitans* and *Porphyromonas gingivalis*, as well as for baker’s yeast, *Saccharomyces cerevisiae*. Uroporphyrin, 7-carboxylporphyrin, 6-carboxylporphyrin, coproporphyrin, and protoporphyrin IX were identified in the investigated microorganisms, and it was shown that the porphyrin profile differs between the two bacteria, as well as for *S. cerevisiae*. To our knowledge, this is the first time the porphyrin profile has been determined for the bacterium *A. actinomycetemcomitans*.

**Electronic supplementary material:**

The online version of this article (doi:10.1007/s00216-015-8864-2) contains supplementary material, which is available to authorized users.

**Graphical Abstract:**
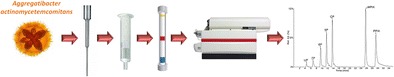
Determination of porphyrins in oral bacteria.

## Introduction

Porphyrins are a group of macromolecules involved in the biosynthesis of several essential biological molecules such as heme and chlorophyll [1, 2]. The basic structure for all porphyrins is porphine, a tetrapyrrolic ring linked by methine bridges. The individual porphyrins are specified by the side chain substituents including alkyl, alkene, or carboxylic acid groups. Owing to their aromatic nature, porphyrins act as pigments that are fluorescent with characteristic bands of absorbance between λ = 390–425 nm [3], a strong Soret band in the λ = 380–500 nm region, and weaker Q-bands in the λ = 500–750 nm region.

Porphyrins have been determined in a variety of matrices, including blood, urine, feces, and also in bacteria [4–7]. The commonly used method for separation of porphyrins is reversed phase high-performance liquid chromatography (RP-HPLC) using methanol or acetonitrile as the organic phase modified with a buffer such as ammonium acetate-acetic acid [2, 5, 8–10]. The fluorescent nature of the porphyrins is most frequently used for detection [6, 8, 11], but mass spectrometry (MS) has also been used in a number of studies [3–5]. Determination of porphyrins in bacteria has mostly been performed using fluorescence detection, utilizing the similarities of the fluorescence spectral properties of porphyrins [7, 12, 13]. This means that detection of porphyrins with fluorescence is a nonselective method. However, when determining porphyrins in biological samples, the number of porphyrin species and the complex nature of the matrix demand a selective detection technique. This can be achieved by using tandem mass spectrometry.

Detection of oral biofilm and early signs of caries can be made with fluorescence techniques, and dental biofilm can be visualized as red fluorescence when the teeth are illuminated with light in the blue spectral region [14–16]. Studies have suggested that obligate anaerobic bacteria are responsible for this red fluorescence because of their increasing numbers in the mature oral biofilm [16, 17]. In a previous study, our research group has shown that also isolated colonies of *Aggregatibacter actinomycetemcomitans* are capable of emiting red fluorescence when exposed to blue light (λ = 370 nm) and suggested that this fluorescence emission is due to endogenously produced porphyrins [18]. The porphyrin content of the bacterium *Propionibacterium acnes* has been investigated in several studies [12, 13, 19, 20], but little work has been done on oral pathogens. Soukos et al. [7] have analyzed the porphyrin content of oral black-pigmented bacteria *Porphyromonas gingivalis*, *Prevotella intermedia*, *Prevotella nigrescens*, and *Prevotella melaninogenica* using RP-HPLC with fluorescence detection.

Studies have demonstrated that bacteria containing porphyrins are sensitive to visible light, in the blue as well as the red spectral region [21, 22]. It has been proposed that oral bacteria can be eradicated using light in the blue region of the visible spectrum, involving a mechanism that is supposed to be based on optical excitation of the bacteria’s endogenous porphyrins [23]. This is used in phototherapy methods (i.e., treatment by visible light without any added exogenous photosensitizer). Since bacterial resistance to antibiotics is increasing, there is a growing interest for alternative treatments of microbial infections. For this reason, it would be of great interest to investigate the content of porphyrins in oral pathogens associated with periodontal disease as well as in oral biofilm, in order to connect bacterial susceptibility to phototoxicity with their content of porphyrins.

The aim of this study was to develop a method for the sensitive and selective determination of porphyrins in oral pathogenic bacteria by combining an efficient extraction and clean-up method for oral bacteria with efficient separation on RP-HPLC and selective detection with mass spectrometry using selected reaction monitoring (SRM). *A. actinomycetemcomitans* and *P. gingivalis* were selected as model oral pathogens since they are periodontopathogenic, non-fastidious anaerobic bacteria that are comparably easy to culture. *Saccharomyces cerevisiae* was used as an additional model organism for validation of the method.

## Material and methods

### Chemicals and reagents

HPLC grade methanol and acetonitrile were purchased from Rathburn Chemicals Ltd. (Walkerburn, Scotland). Formic acid (≥98 %), acetic acid (≥99.8 %), and hydrochloric acid (≥37 %) were obtained from Sigma Aldrich (Schnelldorf, Germany). Deionized water at 18 Ω was produced by a Synergy 185 water purification system from Millipore (Molsheim, France). Ammonium acetate, sodium bicarbonate, and tris(hydroxymethyl)aminomethane (Tris) were all of pro analysi grade and obtained from Merck (Darmstadt, Germany). Dimethylformamide (DMF) was obtained from VWR International (Fount-Sous-Bois, France) and ethylenediaminetetraacetic acid (EDTA) disodium dihydrate salt of reagent grade from Scherlab S.L. (Sentmenat, Spain). *S. cerevisiae* (commonly known as Baker’s yeast) was used for method validation. It was obtained from Jästbolaget (Sollentuna, Sweden).

**Fig. 1:**
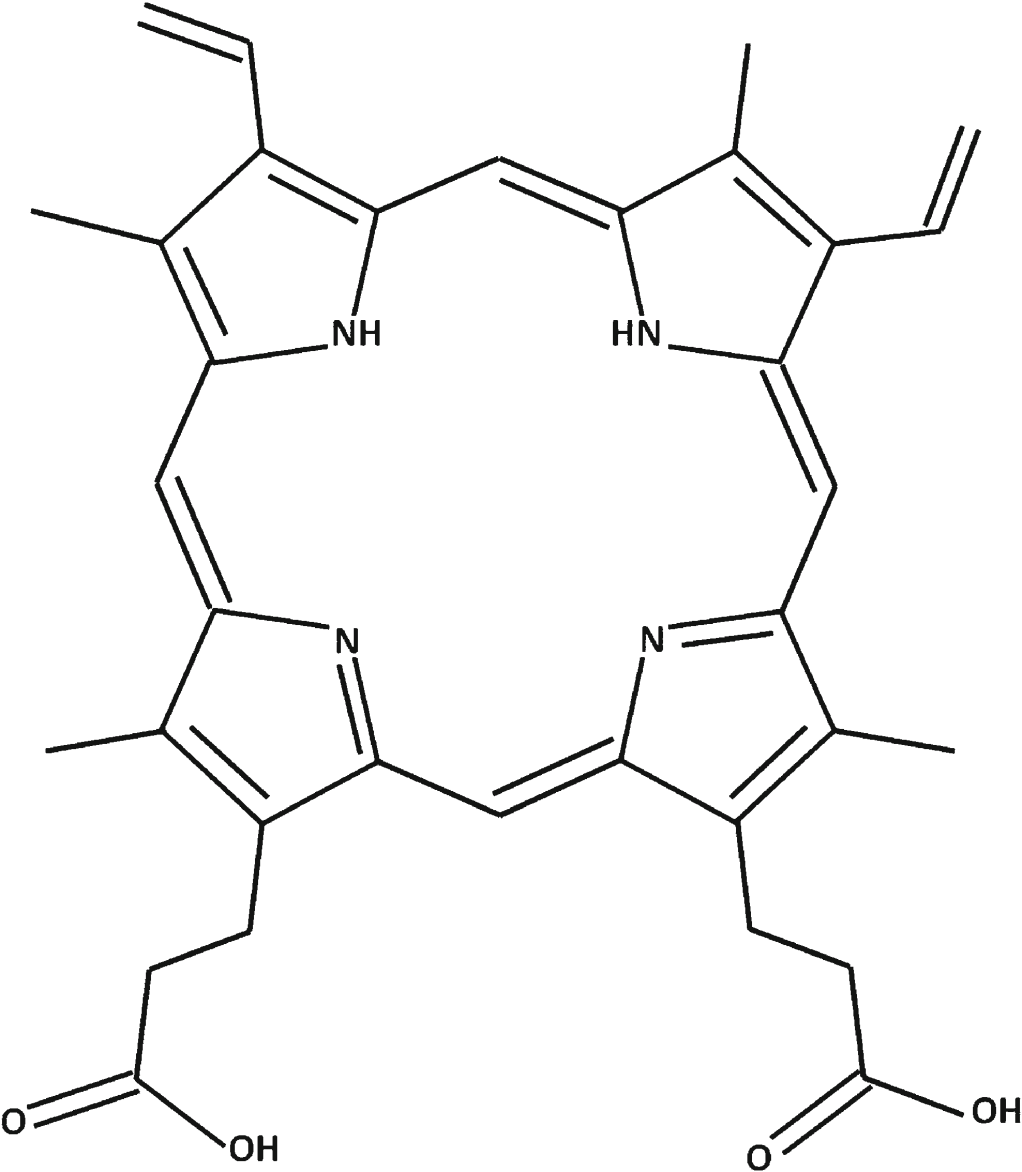
Structure of protoporphyrin IX (3,7,12,17-tetramethyl-8,13-divinyl-2,18-porphinedipropionic acid)

Porphyrin standards were purchased as individual compounds and as a chromatographic marker kit (Table 1) from Frontier Scientific, Logan, UT, USA (part No. CMK-1A). The structure of protoporphyrin IX is shown in Fig. 1.

**Table 1:** Abbreviations, CAS no, purities, molecular weights, and manufacturers for the investigated porphyrins

Compound	Abbreviation	CAS no.	Purity (%)	Mw (Da)	Manufacturer
Uroporphyrin I	UP I	607-14-7	≥90	830.75	Frontier Scientific Inc.
7-Carboxylporphyrin I	7P I	65406-45-3	≥90	786.74	Frontier Scientific Inc.
6-Carboxylporphyrin I	6P I	73913-56-1	≥90	742.73	Frontier Scientific Inc.
5-Carboxylporphyrin I	5P I	28100-66-5	≥90	698.72	Frontier Scientific Inc.
Coproporphyrin I	CP I	531-14-6	≥90	654.71	Frontier Scientific Inc.
Coproporphyrin III	CP III	14643-66-4	≥97	654.71	Frontier Scientific Inc.
Mesoporphyrin IX	MP IX	493-90-3	≥90	566.69	Frontier Scientific Inc.
Mesoporphyrin IX dihydrochloride	MP IX Cl	68938-72-7	95	639.61	Sigma Aldrich
Protoporphyrin IX	PP IX	553-12-8	≥95	562.66	Sigma Aldrich
Hemin	Hemin	16009-13-5	≥90	651.94	Sigma Aldrich

### Preparation of solutions and standards

A 1 M ammonium acetate buffer was prepared by dissolving ammonium acetate in deionized water and adjusting pH to 5.16 by addition of acetic acid. TE-buffer was made by mixing a 1 M solution of Tris with a 0.1 M EDTA (pH 8) solution to give concentrations of 5 mM Tris and 10 mM EDTA adjusting the pH to 7.2 using HCl.

The chromatographic marker kit was dissolved in 6 M formic acid to obtain a stock solution with a concentration of 2 μmol/L for each of uroporphyrin I (UPI), 7-carboxylporphyrin I (7PI), 6-carboxylporphyrin I (6PI), 5-carboxylporphyrin I (5PI), coproporphyrin I (CPI), and mesoporphyrin IX (MPIX). Separate stock solutions with concentrations between 0.2 and 0.3 mmol/L were prepared of MPIX, protoporphyrin IX (PPIX), and coproporphyrin III (CPIII). This was made in DMF:methanol 1:1 (*v*/*v*) because of the higher hydrophobicity of these compounds. Working standards were prepared in 6 M formic acid from the stock solutions. Stability tests in 6 M formic acid showed that the more hydrophobic compounds PPIX and MPIX were unstable in a polar solvent (data not shown), which made daily preparation of working standards necessary. This instability is probably due to the aggregation of the porphyrins in aqueous solution [24].

### Culturing and harvesting bacteria

*A. actinomycetemcomitans* strain ATCC 33384 serotype c and *P. gingivalis* strain ATCC BAA-308/W83, both obtained from the Department of Clinical Bacteriology at the University of Gothenburg, were cultivated in a CO_2_ incubator, model T303 (Assab Medicine, Stockholm, Sweden). *A. actinomycetemcomitans* was grown in a 4.9 % CO_2_ atmosphere and *P. gingivalis* was grown under anaerobic conditions, both at a temperature of 37.1 °C. *A. actinomycetemcomitans* was grown on Columbia agar with 5 % defibrinated horse blood and *P. gingivalis* on Brucella agar containing horse blood, hemin, and K-vitamin (Substrate unit, Karolinska University Hospital, Huddinge). Subculturing was made by incubation for 4 d on blood agar plates followed by transfer to new blood agar plates and grown for an additional 7 d for *A. actinomycetemcomitans* and 12 d for *P. gingivalis*. The bacteria were harvested from the agar plates using a sterile plastic cell scraper and transferred to 500 μL of 0.4 % NaCl in a 1.5 mL Eppendorf vial and the sample size measured gravimetrically.

### Sample preparation

The harvested bacterial cell material was transferred to a test tube, vortexed for 30 s, and pelleted by centrifugation for 10 min at 2500 *g*. The supernatant was removed, 1000 μL of TE-buffer was added, and the pellet was resuspended by vortexing. The test tube was protected from light by wrapping in aluminum foil and put to digest for 1 h at room temperature, after which proteins were precipitated by addition of 1000 μL of 6 M formic acid. The cells were then further lysed using an ultrasonication rod for 1 min. The sample was subsequently centrifuged for 10 min at 2500 *g* and the supernatant containing porphyrins was cleaned up using an end-capped Isolute C18 SPE- cartridge (3 mL, 200 mg; International Sorbent Technology Ltd., Hengoed, UK). The SPE cartridge was preconditioned with 3 mL of methanol:acetonitrile 9:1 (*v*/*v*), 3 mL of deionized water, and 3 mL of ammonium acetate (1 M, pH 5.16). The sample was added and washed with 3 mL of ammonium acetate (1 M, pH 5.16). Porphyrins were eluted using 2 mL of acetone:formic acid, 9:1 (*v*/*v*). The eluent was placed in a heated water bath at 70 °C and slowly evaporated to dryness under a gentle stream of nitrogen. Finally the volume was adjusted to 500 μL using 6 M formic acid.

### Instrumentation

Two HPLC systems were used for analysis of the porphyrins. The first system consisted of an HPLC system from Perkin Elmer (Norwalk, CT, USA) with a binary solvent delivery system consisting of two 200 series micro pumps, a 200 series autosampler equipped with a 5 μL injection loop, and a 200 series vacuum degasser, and were used for the analysis of *A. actinomycetemcomitans*. The second system was an Agilent HPLC system (Wilmington, DE, USA) consisting of an 1100 binary pump solvent delivery system, an 1100 degasser, and an 1100 autosampler. The latter system was used for analysis of *P. gingivalis* and *S. cerevisiae* using 5 μL as injection volume. Both HPLC systems were coupled to a SCIEX API 2000 triple quadrupole mass spectrometer (Toronto, ON, Canada) equipped with a TurboIon Spray interface run in electrospray ionization mode. Analyst 1.4.2 software from SCIEX was used for instrument control and data processing.

Separation was performed on an ACE 3 C18-PFP column (75 × 2.1 mm, d_p_ = 3 μm, Advanced Chromatography Technologies Ltd., Aberdeen, Scotland) with a C18-PFP guard column (2.1 mm i.d.) and a mobile phase flow rate of 100 μL/min. Mobile phase A consisted of 95 % water, 5 % acetonitrile, and 0.1 % formic acid (*v*/*v*/*v*), and mobile phase B 95 % acetonitrile, 5 % water, and 0.1 % formic acid (*v*/*v*/*v*). A linear gradient program was applied, increasing the mobile phase B from 30 to 50 % during the first 10.0 min, followed by a linear increase of mobile phase B up to 100 % from 10.0 to 10.2 min. The mobile phase was held at 100 % B for another 24.8 min. Before each analysis, the column was equilibrated for 20.0 min with 30 % mobile phase B.

Detection of porphyrins was made in positive electrospray ionization mode (ESI+) and SRM with three compound-specific product ions for each porphyrin. Porphyrins were identified by retention time and the presence of all three product ions. All transitions were used for quantification. The instrument was tuned by direct infusion of standard solution with a concentration of 2 nmol/mL of each of the porphyrins in 6 M formic acid solution. MS parameters were as follows: ion source temperature 200 °C, capillary voltage 5 kV, nebulizer gas (N_2_) 35 psi, collision gas (N_2_) 8 psi, and curtain gas (N_2_) 20 psi. The MS/MS parameters used for analysis can be seen in Table 2.

**Table 2:** Conditions for the MS/MS SRM analyses

Porphyrin	[M+H]^+^	Time window	Transition 1			Transition 2			Transition 3		
	*m*/*z*	(min)	*m*/*z*	CE (V)^a^	CXP (V)^b^	*m*/*z*	CE (V)^a^	CXP (V)^b^	*m*/*z*	CE (V)^a^	CXP (V)^b^
Uroporphyrin I	831	0.0–5.5	727	70	22	623	85	22	655	85	22
7-Carboxylporphyrin I	787	5.5–8.2	683	85	22	670	85	22	623	85	22
6-Carboxylporphyrin I	743	8.2–11.0	639	70	22	507	90	22	521	90	22
5-Carboxylporphyrin I	699	11.0–13.2	463	95	20	595	119	22	640	47	24
Coproporphyrin I	655	13.2–19.5	537	65	24	596	51	26	523	63	26
Mesoporphyrin IX	567	19.5–27.5	449	61	24	479	85	22	508	43	24
Protoporphyrin IX	563	27.5–35.0	445	65	22	504	45	22	489	89	22

^a^ Collision energy

^b^ Collision exit potential

### Determination of microbial killing efficiency

*A. actinomycetemcomitans* was grown and subcultured under the conditions described above. The bacteria were harvested at d 4 of incubation, transferred to a test tube containing 1 mL of 0.4 % NaCl, and vortexed for 30 s giving a stock solution of bacteria. One hundred microliters of the stock solution were added to 900 μL of 0.4 % NaCl and vortexed, resulting in a dilution factor of 10^–1^. This dilution process was successively repeated, giving seven control samples with dilution factors ranging from 10^–1^ to 10^–7^. From each dilution step, 100 μL were evenly distributed with a sterile glass spreader on an agar plate. The remaining 900 μL of the stock solution were put in TE-buffer and treated with formic acid and ultrasonication as described above. Same dilution procedure as described above was performed and 100 μL were put on agar plates. All agar plates were incubated for 4 d and plates containing between 30 and 300 colony-forming units (CFU) were counted to estimate the killing efficiency. The experiment was performed in triplicate for statistical evaluation.

### Linearity, LOD, and LOQ

Calibration standards containing UPI, 7PI, 6PI, 5PI, CPI, MPIX, and PPIX were prepared at six concentration levels: 50, 100, 300, 500, 700, and 900 pmol/mL in 6 M formic acid. All calibration standards were injected in triplicate. The resulting peak area (*y*) versus concentration (*x*) was plotted for each porphyrin and treated by least squares method of linear regression.

For determination of LOD and LOQ, the signal to noise ratio (S/N) was calculated from injections of 25 fmol of each porphyrin in standard solution. The noise was defined as the standard deviation of the peak area and the signal was defined as the mean area of triplicate injection. LOD and LOQ were defined as three and 10 times the S/N ratio, respectively.

### Accuracy, precision, and recovery

Accuracy was determined by analyzing triplicate samples of spiked extracts from *S. cerevisiae* with a known concentration of each porphyrin. It was calculated by the relative difference between the mean of the measured concentration and the true concentration. Endogenously produced amounts of porphyrins in the extracts were subtracted from the spiked samples. MPIX was used as a volumetric internal standard (IS_vol)_) to correct for differences in injection volume and/or ionization efficiency between analytical runs. Spiked extracts from *S. cerevisiae* were analyzed in triplicate during the same day to investigate the intra-day precision of the analysis and is presented as coefficient of variation (CV%).

For estimation of recovery, a standard solution containing 0.5 nmol/mL of each porphyrin in 6 M formic acid was put through the sample preparation steps described above. Detector responses from the standard mixture going through the clean-up steps were compared with the detector responses from the untreated standard solution. The experiment was performed in triplicate.

## Results and discussion

### HPLC separation of porphyrins

**Fig. 2:**
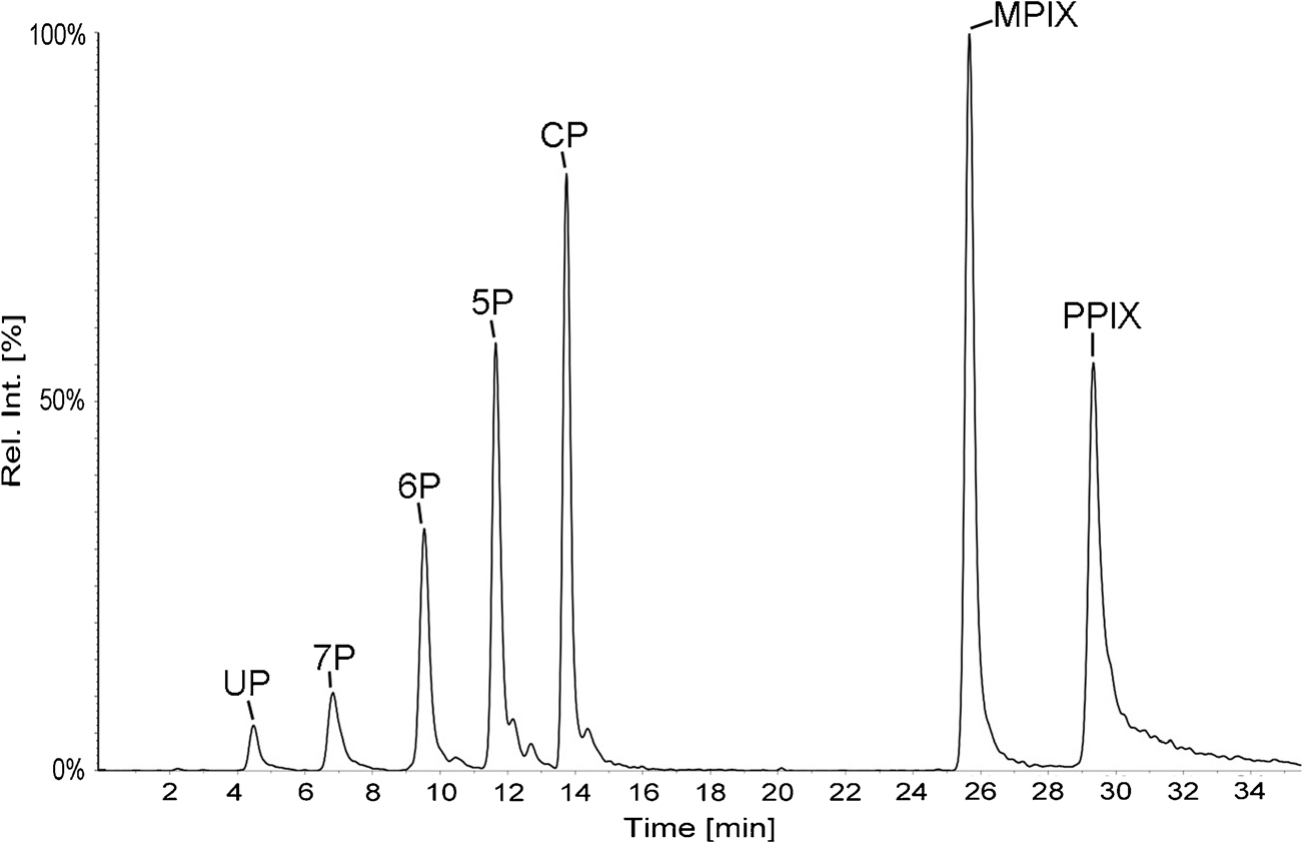
Chromatogram from the HPLC/MS/MS analysis of a porphyrin standard mixture. UP: uroporphyrin I; 7P: 7-carboxylporphyrin I; 6P: 6-carboxylporphyrin I; 5P: 5-carboxylporphyrin I; CP: coproporphyrin I; MPIX: mesoporphyrin IX; PPIX: protoporphyrin IX

In Fig. 2, a chromatogram is shown that demonstrates the separation of a standard solution containing the seven porphyrins. They are all well separated with a resolution factor (R) higher than 1.5 (i.e., baseline separation) with a total run time of 30 min. The elution order is UPI, 7PI, 6PI, 5PI, CPI, MPIX, and PPIX, corresponding to the decreasing number of carboxylic acid groups attached to the basic porphine structure. All porphyrins have a number of possible isomers of which type I and III are the ones commonly present. Type III isomers are formed by enzymatic action, whereas type I isomers are formed by spontaneous chemical ring closure in the absence of enzymes [3]. The separations of the type I isomers (UPI, 7PI, 6PI, and 5PI), type IX isomers of MPIX and PPIX, and both type I and type III of CP were evaluated. CP type III isomer elutes after type I and the isomers are well separated with R > 1.5. CPIII has not been validated with this method but has been assumed to have identical properties to CPI.

### Detection of porphyrins using ESI MS/MS

There are large differences in the ESI+ ionization efficiency between the porphyrins. UPI contains eight carboxylic acid substituents, whereas PPIX contains only two. This could be a possible reason for the latter compound having a 40 times higher response than UPI in the Agilent/Sciex API 2000 LC/MS system. That the less polar PPIX has a higher response in ESI-MS compared with the more polar porphyrin UPI is most probably due to the various distributions of ions of different hydrophobicity in the droplets of the spray [25]. In the Perkin Elmer/Sciex API 2000 LC/MS system, the difference in response between the porphyrins was substantially lower. The retention times were different in the two systems, which meant that PPIX was ionized in different mobile-phase compositions in the two LC/MS systems (Figs. 5 and 6). Selection of mobile phases has shown to be important when ESI is used. Ionization efficiency is influenced by the solvent composition where high water content of the mobile phase tends to give decreased ionization efficiency. This is due to the high viscosity of water, leading to difficulties in forming a stable spray, whilst a high organic content results in a better aerosol formation. This effect can be seen when compounds of varying polarity are analyzed with gradient elution in RP-HPLC. Thus, there are no straight-forward conclusions that can be drawn regarding the differences in ESI ionization efficiency of the porphyrins observed in this study.

### Determination of microbial killing efficiency

The agar plates with nontreated bacteria showed a CFU range between 30 and 300 at dilution factors 10^–3^ to 10^–4^. The agar plates on which the TE-buffer and ultrasonication treated bacteria were cultivated the CFU count was zero in all dilution steps, yielding a bacterial killing efficiency ≥99.98 %. The lysis of bacteria is efficient using this method and it is thus assumed that most of the porphyrins are extracted from the bacteria. *A. actinomycetemcomitans* is a gram-negative bacterium, which means that it has an inner and an outer cell membrane, making the bacterium hard to lyse. High lysis efficiency of this bacterium indicates that the method will yield a good porphyrin extraction for other gram-negative, gram-positive and yeast cells as well.

### Method validation

#### Linearity, LOD, and LOQ

**Fig. 3:**
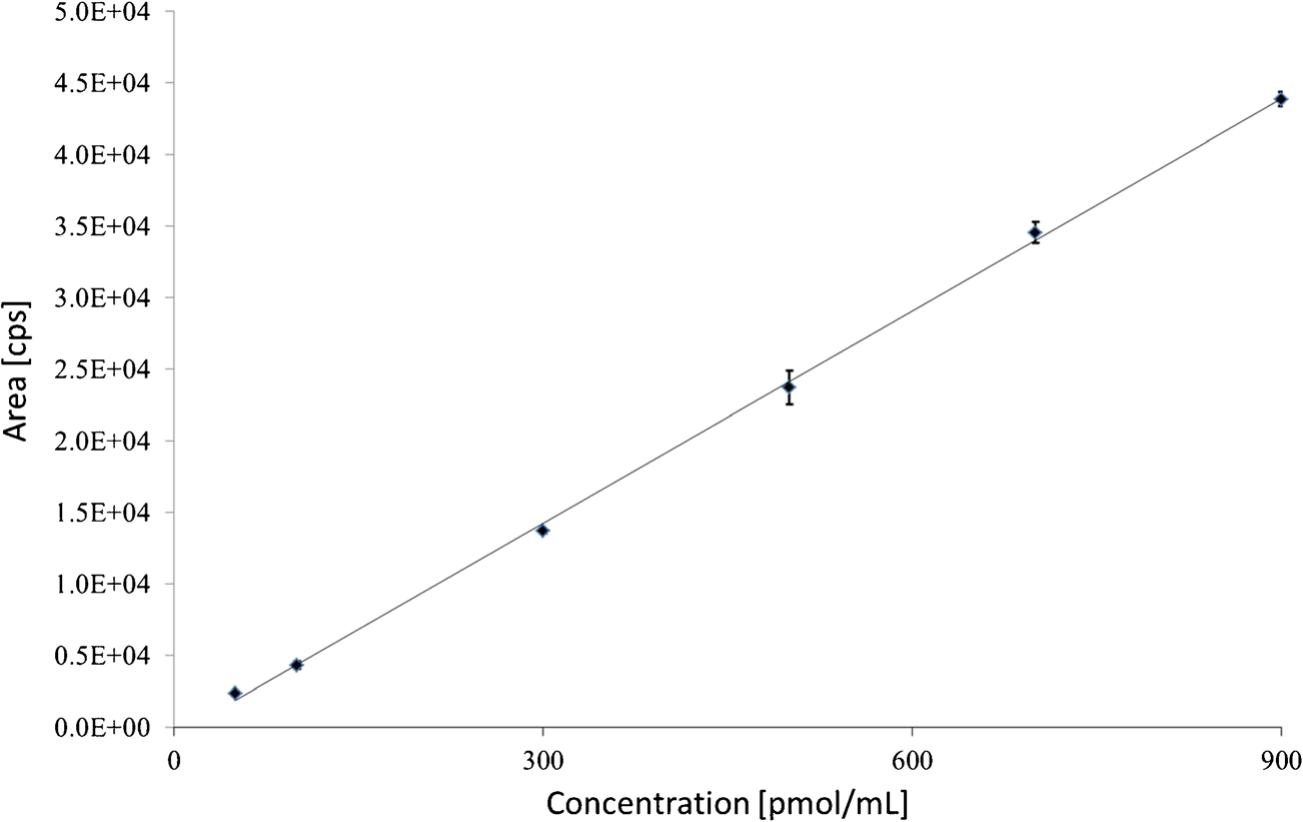
Calibration curve for CPI

The linearity of the calibration curves was high for all the investigated porphyrins demonstrated by coefficients of determination (R^2^) being higher than 0.98 for all the analytes. In Table S1 in the Electronic Supplementary Material (ESM) the linear regression equation and R^2^ for each porphyrin are listed, and the calibration curve for CPI is shown in Fig. 3.

Coefficients of variation (CV%) were calculated for retention time and peak area (A) at each concentration level (*n* = 18). CV% for area units are mean values from the average at each concentration level in the calibration curve and are listed in ESM Table S2. Retention times showed high stability with CV% ≤1 % for all analytes. The mean CV% in area at six concentration levels was <10 % for all porphyrins and as low as 3 % for MPIX.

S/N ratio, LOD, and LOQ for each porphyrin are summarized in Table 3. LOD ranged between 2.7 and 11.8 fmol (1.5–8.8 pg) for all porphyrins. Lowest LOD, 2.7 fmol (1.5 pg), was demonstrated by PPIX.

**Table 3:** S/N, LOD and LOQ calculated using an injected amount of 25 fmol of each porphyrin (*n* = 3)

Porphyrin	S/N ratio	LOD [fmol injected,( ±SD)]	LOQ [fmol injected, (±SD)]
Uroporphyrin I	9.4	7.9 (±0.8)	26.5 (±2.8)
7-Carboxylporphyrin I	6.8	11.1 (±1.6)	36.9 (±5.5)
6-Carboxylporphyrin I	6.3	11.8 (±1.9)	39.4 (±6.2)
5-Carboxylporphyrin I	19.5	3.8 (±0.2)	12.8 (±0.7)
Coproporphyrin I	10.2	7.3 (±0.7)	24.5 (±2.4)
Mesoporphyrin IX	11.0	6.8 (±0.6)	22.8 (±2.1)
Protoporphyrin IX	27.6	2.7 (±0.1)	9.1 (±0.3)

#### Accuracy, precision, and recovery

Accuracy, precision, and recovery of the complete analytical method for the investigated porphyrins are summarized in Table 4. Intra-day precision was high (RSD <5 %) for all porphyrins, including the internal standard compound. Accuracy was also good, demonstrated by a deviation from the reference value of less than 7 % for four of the porphyrins. For the two “extreme” compounds (i.e., those with eight (UPI) and two (PPIX) carboxylic acid substituent groups), the deviation was 13 and 19 %, respectively. The recoveries for the porphyrins through the entire sample preparation were high, between 79 and 99 % for four of the porphyrin analytes as well as for the internal standard, and also showing a high reproducibility (RSD ≤6 %) for these compounds. Also regarding recoveries, the results for the two extreme compounds, UPI and PPIX, deviated from the other analytes. These two porphyrins showed lower recoveries, 67 and 55 % respectively, with slightly lower reproducibility, 9 and 16 %, respectively. Since this group of porphyrins varies widely in polarity, having between two to eight carboxylic acid substituent groups and also a large variation in pKa, it is difficult to obtain a short and efficient method with high recoveries optimal for all the individual porphyrins. The described method does, however, demonstrate a good accuracy with high reproducibility, as well as good recoveries for most compounds with high reproducibility for all the analytes. By investigating the individual parts of the method it was clear that no degradation of porphyrins was caused by the TE digestion or the ultrasonication. The decrease in recovery of the analytes was solely due to the SPE clean-up. This is quite plausible, considering the very large difference in polarity and pKa values of these analytes yielding a broad elution profile from the SPE.

**Table 4:** Accuracy, intra-day precision, and recovery (*n* = 3)

Porphyrin	Accuracy [%, (±SD)]	Intra-day precision [%]	Recovery [%, (±SD)]
Uroporphyrin I	113 (±14)	3	67 ( ±6)
7-Carboxylporphyrin I	93 (±1)	1	79 ( ±2)
6-Carboxylporphyrin I	99 (±6)	3	88 ( ±5)
5-Carboxylporphyrin I	97 (±7)	5	84 ( ±2)
Coproporphyrin I	99 (±4)	2	99 ( ±3)
Mesoporphyrin IX	IS_vol_	4	79 ( ±1)
Protoporphyrin IX	81 (±1)	5	55 ( ±9)

#### Matrix effects

Evaluation of matrix effects is essential for all MS methods since interfering substances in the sample can alter the response leading to ion suppression or ion enhancement. *S. cerevisiae* was used as a model matrix to determine matrix effects of residual cell material in the sample analysis as it is often used as a test organism in chromatographic evaluations [26–29]. Three samples of *S. cerevisiae* (approximately 60 mg per sample) were put through the sample preparation steps, pooled, and spiked with known amounts of UPI, 7PI, 6PI, 5PI, CPI, CPIII, MPIX, and PPIX prior to analysis. Matrix effects were calculated by comparing the response factors for the porphyrins in the spiked extract with those obtained from standard prepared in 6 M formic acid. Endogenously produced amounts of porphyrins in the extracts were subtracted from the spiked samples. The mean matrix effects for the individual porphyrins were UPI –5 %, 7PI 9 %, 6PI –1 %, 5PI 2 %, CPI +13 %, CPIII –1 %, MPIX –4 %, and for PPIX –14 %. This demonstrates that the matrix effect from residual cell material is low for all the investigated porphyrins using this method.

### Porphyrin content in *A. actinomycetemcomitans*, *P. gingivalis*, and *S. cerevisiae*

**Fig. 4:**
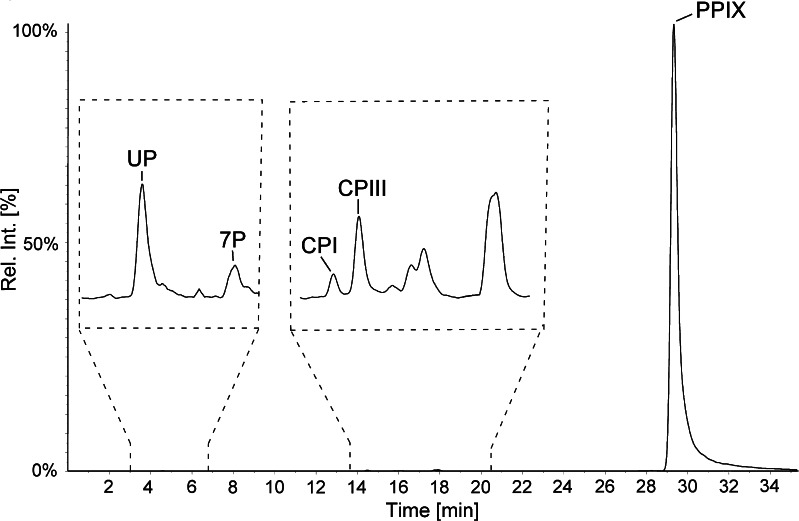
HPLC-MS/MS chromatogram of porphyrins extracted from *A. actinomycetemcomitans* cultivated at 7 d. Parts of chromatogram in dashed boxes are enlarged for better visual effect. Analytes present in the sample: UP (184 ng/g), 7P (28 ng/g), CPI (6 ng/g), CPIII (20 ng/g), and PPIX (286 ng/g)

*A. actinomycetemcomitans* was cultivated on five agar plates and pooled to get an average of endogenously produced porphyrins. Extracts of *A. actinomycetemcomitans* were analyzed the same day using the described method and porphyrins were identified through retention time and three characteristic SRM transitions in the MS/MS analysis. A typical chromatogram from bacterial sample analysis is shown in Fig. 4, identifying UP, 7P, CPI, CPIII, and PPIX. MPIX is not an intermediate in biosynthesis of heme and could not be detected in any of the analyzed bacteria extracts. This makes it suitable for use as an internal standard. Highest concentrations were PPIX and UP, with concentrations of 286 and 184 ng/g respectively; 7P, CPI, and CPIII were of lower concentrations with 28, 6, and 20 ng/g, respectively.

**Fig. 5:**
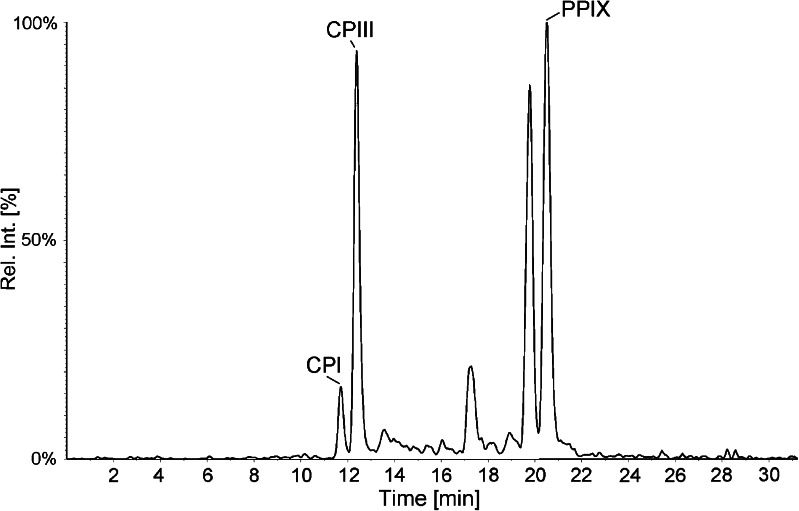
HPLC-MS/MS chromatogram of porphyrins extracted from *P. gingivalis* cultivated for 12 d.. Three porphyrins were detected in the sample: CPI (4 ng/g), CPIII (26 ng/g), and PPIX (214 ng/g)

*P. gingivalis* were cultivated anaerobically on five agar plates and pooled. CPI, CPIII, and PPIX were detected and quantified in bacterial extracts, Fig. 5. PPIX had clearly the highest concentration with 214 ng/g, whereas CPI and CPIII had lower concentrations with 4 and 26 ng/g, respectively. Both CP and PPIX have been detected earlier in *P. gingivalis* [7, 30]. The concentration of porphyrins are in the same range as were found in *A. actinomycetemcomitans* with the exception of UP and 7P not being detected. *P. gingivalis* lacks all of the enzymes involved in the formation of 5-aminolaevulinic acid (porphyrin precursor) and most of the enzymes involved in the biosynthesis of porphyrins and heme, which are all present in *A. actinomycetemcomitans* and, thus, it is expected that more porphyrins will be found in the latter. *P. gingivalis* does, however, have the enzyme for conversion of CPIII to PPIX as well as ferrochelatase for formation of heme [31]. *P. gingivalis* is, therefore, dependent on an external source of iron and is able to utilize iron by uptake of organic iron including hemin, hemoglobin, myoglobin, cytochrome *c*, transferrin, as well as inorganic iron in the form of ferric chloride and ferric nitrate [32]. It has been shown that the heme captured on the cell surface are porphyrin-specific, and porphyrins with similar structures to PPIX, including MPIX and deuteroporphyrin IX, bind to the receptors by low affinity interactions [33, 34]. The bacteria could have an uptake of porphyrins from the growth medium, and this can be an explanation as to why CPI and CPIII were detected in *P. gingivalis* despite the fact that they lack enzymes for synthesis of coproporphyrin isomers, but this specific uptake has not been studied. Another explanation could be that *P. gingivalis* has an alternative pathway of heme synthesis similar to that observed in *Desulfovibrio vulgaris* where the classic route is bypassed but rejoined at CPIII [35, 36].

**Fig. 6:**
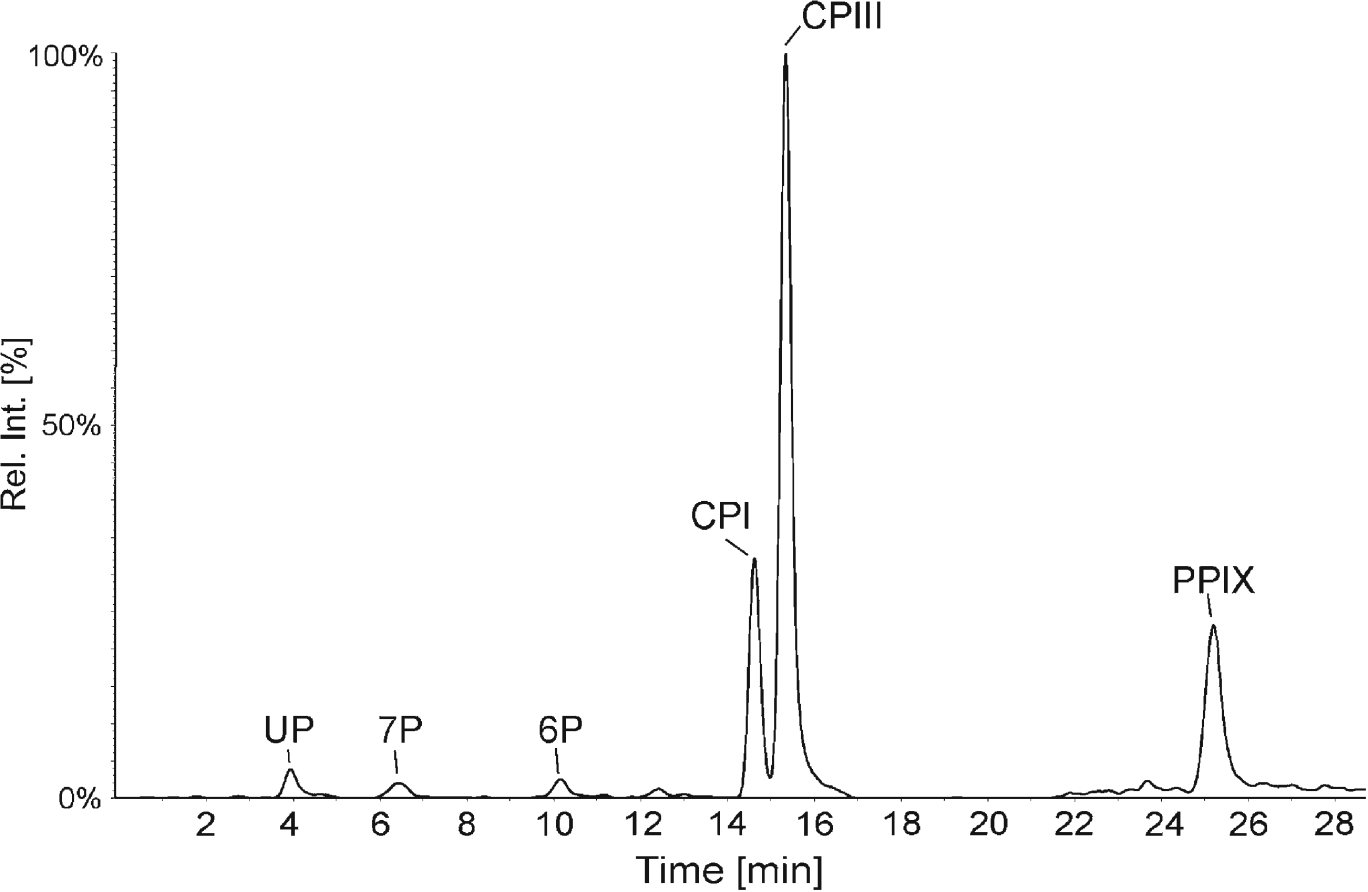
HPLC-MS/MS chromatogram of porphyrins extracted from *S. cerevisiae*. Six porphyrins were detected in the sample: UP (19 ng/g), 7P (5 ng/g), 6P (5 ng/g), CPI (11 ng/g), CPIII (79 ng/g), and PPIX (2 ng/g)

When *S. cerevisiae* was analyzed, a large number of porphyrins were detected: UP, 7P, 6P, CPI, CPIII, and PPIX. PPIX was the least abundant porphyrin with a concentration of only 2 ng/g, while on the other hand it was the predominant porphyrin in *A. actinomycetemcomitans* and *P. gingivalis*. The most abundant porphyrin in *S. cerevisiae* was CPIII with a concentration of 79 ng/g. A chromatogram from the LC/MS analysis is shown in Fig. 6. Concentrations and percent of porphyrin content for all microbes are summarized in Table 5.

**Table 5:** Concentrations of porphyrins in extracts of the oral bacteria *A. actinomycetemcomitans* and *P. gingivalis*, and baker’s yeast *S. cerevisiae*

Organism	*A. actinomycetemcomitans*		*P. gingivalis*		*S. cerevisiae*	
Porphyrin	Concentration [ng/g]	% of total porphyrin content	Concentration [ng/g]	% of total porphyrin content	Concentration [ng/g]	% of total porphyrin content
Uroporphyrin	184	35	n.d.	–	19	16
7-Carboxylporphyrin	28	5	n.d.	–	5	5
6-Carboxylporphyrin	n.d.	–	n.d.	–	5	4
5-Carboxylporphyrin	n.d.	–	n.d.	–	n.d	–
Coproporphyrin I	6	1	4	2	11	9
Coproporphyrin III	20	4	26	11	79	65
Mesoporphyrin IX	n.d.	–	n.d.	n.d	n.d	–
Protoporphyrin IX	286	55	214	87	2	1
Sum:	524	100	244	100	121	100

*n.d*. not detected

## Conclusions

A method for extraction and analysis of porphyrins from bacteria and yeast has been developed and validated. *A. actinomycetemcomitans* and *S. cerevisiae* were used for evaluating matrix effects. Highly selective detection using MS/MS of porphyrins in oral bacteria was applied, and the method offers the possibility of accurate characterization of bacterial porphyrin contents. All compounds were well separated, including the isomers CPI and CPIII, using a PFP modified C18 stationary phase for the HPLC separation. The extraction method is good with efficient cell lysis and high recoveries and reproducibility. Sample clean-up using SPE gave minimal matrix effects for all investigated porphyrins. The method was applied to *P. gingivalis* for determination of porphyrins, showing that the method can be successfully extended for use on other oral bacteria. In *P. gingivalis*, CPI, CPIII, and PPIX were identified, in consistent with earlier studies. For the first time, to our knowledge, UP, 7P, CPI, CPIII, and PPIX were identified in *A. actinomycetemcomitans*.

## Electronic supplementary material

ESM 1 (PDF 538 kb)

## Abbreviations

5PI: Pentacarboxylic porphyrin I
6PI: Hexacarboxylic porphyrin I
7PI: Heptacarboxylporphyrin I
CFU: Colony forming units
CPI: Coproporphyrin I
CPIII: Coproporphyrin III
MPIX: Mesoporphyrin IX
PPIX: Protoporphyrin IX
S/N: Signal to noise
SRM: Selected reaction monitoring
TE: Tris/EDTA
UPI: Uroporphyrin I
