# Supplementary material for: Determination of porphyrins in oral bacteria by liquid chromatography electrospray ionization tandem mass spectrometry
Source: Anal Bioanal Chem. 2015 Jul 14;407(23):7013–23. doi: 10.1007/s00216-015-8864-2 (PMC4551553; doi:10.1007/s00216-015-8864-2)
Supplement: Supplementary file 1 — (PDF 538 kb) [file 216_2015_8864_MOESM1_ESM.pdf]

Analytical and Bioanalytical Chemistry

Electronic Supplementary Material

Determination of porphyrins in oral bacteria by liquid chromatography electrospray ionization tandem mass spectrometry

Jonas Fyrestam, Nadja Bjurshammar, Elin Paulsson, Annsofi Johannsen, Conny Östman

Table S1 Linear regression equation and R2 values for porphyrins using a calibration curve for selected porphyrins between 50-900 pmol/mL

Porphyrin	Linear regression equation	Coefficient of determination R^2
Uroporphyrin I	Y = 27 x	0.995
7-carboxylporphyrin I	Y = 70 x	0.987
6-carboxylporphyrin I	Y = 190 x	0.999
5-carboxylporphyrin I	Y = 380 x	0.998
Coproporphyrin I	Y = 490 x	0.999
Mesoporphyrin IX	Y = 830 x	0.997
Protoporphyrin IX	Y = 1 200 x	0.988

^{Y} = area in cps

^{x} = concentration in pmol/mL

Table S2 CV% for retention time and area at six different concentration levels of the standard solution (n=18)

Porphyrin	Retention time CV%	Area CV%
Uroporphyrin I	1.0	7.3
7-carboxylporphyrin I	0.9	9.4
6-carboxylporphyrin I	0.4	6.2
5-carboxylporphyrin I	0.3	3.4
Coproporphyrin I	0.2	3.6
Mesoporphyrin IX	0.1	2.8
Protoporphyrin IX	0.1	4.3
